# Risk factors and negative consequences of patient’s delay for penile carcinoma

**DOI:** 10.1186/s12957-016-0863-z

**Published:** 2016-04-27

**Authors:** Wen Gao, Le-bin Song, Jie Yang, Ning-hong Song, Xin-feng Wu, Ning-jing Song, Di Qiao, Chen Chen, Jia-yi Zhang, Zeng-jun Wang

**Affiliations:** Department of Urology, First Affiliated Hospital of Nanjing Medical University, Nanjing, 210029 Jiangsu China; Department of Oncology, First Affiliated Hospital of Nanjing Medical University, Nanjing, Jiangsu China; College of Basic Medical Sciences, Nanjing Medical University, Nanjing, Jiangsu China; Institute of Dermatology Surgery, China Academy of Medical Sciences and Peking Union Medical College, Nanjing, Jiangsu China; Department of Dermatology, Shanghai Skin Diseases Hospital, Shanghai, China; Department of Urology, Jiangsu Province Official Hospital, Nanjing, Jiangsu China; State Key Laboratory of Oral Diseases, West China School of Stomatology, Sichuan University, Chengdu, China

**Keywords:** Penile carcinoma, Patient’s delay, Risk factors, Male sexual function, Relapse, Overall survival

## Abstract

**Background:**

Delayed first medical consultation (patient’s delay) is quite common in cases of penile carcinoma (PC), but its reasons and impacts remain unclear. We conducted this study to ascertain risk factors resulting in delayed treatment seeking and evaluate its influence on prognosis.

**Methods:**

From 2004 to 2010 at 4 centers, 254 patients were enrolled into this study from 262 consecutive PC cases. Patients’ sexual performance was investigated using the International Index of Erectile Function (IIEF)-15 at the sixth-month end after treatment. Data for prognostic analyses was obtained via a 5-year follow-up.

**Results:**

A multivariate model ascertained 4 risk factors (single, living in rural areas, heavy drinking alcohol, and aspecific initial symptoms) and 1 protective factor (history of condyloma) significantly associated with patient’s delay. Delay >3 months led to significant risks for adverse clinical characteristics, low penis-sparing rate, and poor sexual function restoration. Although patient’s delay was not found to impact on postoperative relapses and 5-year overall survival (OS), patients with delay >6 months had significantly inferior 2-year OS.

**Conclusions:**

Single, living in rural areas, heavy drinking alcohol, and aspecific initial symptoms are significant risk factors of PC associated with patient’s delay. Delay >3 months will lead to significantly inferior clinical consequences. Minimizing patient’s delay is the key to avoid amputation and retain superior sexual potency. Improving patient education on initial symptoms of PC is necessary in men of >40 years old.

**Electronic supplementary material:**

The online version of this article (doi:10.1186/s12957-016-0863-z) contains supplementary material, which is available to authorized users.

## Background

Today, nearly 80 % of patients with penile carcinoma (PC) can be cured [1]. As more patients achieve long-term survival, sexual dysfunction, poor cosmetic appearance, and disability of upright urination are increasingly recognized as negative consequences that severely affect the quality of life and psychosocial well-being. Although various penis-sparing techniques have been successfully developed to replace devastating amputation as the standard treatment of superficial PC (stages Tis, Ta, and T1a), partial amputation or total penectomy with perineal urethrostomy is still the unique strategy for patients of stages T1b–T4 to acquire a curative result [1, 2]. Therefore, treatment at an early stage of disease is the key for patients to acquire acceptable functional and cosmetic outcomes. Unfortunately, delayed first medical consultation (patient’s delay) is quite common in cases of PC. A prospective study [3] and a retrospective study [4] in Sweden respectively exhibited 65 and 37 % of PC patients in a delay of more than 6 months. The reasons behind this considerable ratio remain largely unclear and might involve multiple sociodemographic and psychological factors as well as aspecific initial symptoms [3, 4].

PC is rare in Europe with an incidence of 0.1 to 0.9/100,000 men [5], but it is still a challenge for the developing world. The incidence reaches 8.3/100,000 in Brazil and even higher in Uganda [1, 6]. The higher morbidity makes independent studies in developing countries, focusing on clarifying the phenomenon of patient’s delay, become more urgent because of the great sociodemographic diversity between developing and developed countries. In addition, few studies have investigated the exact impacts of delay on tumor size and stage, treatment strategies, postoperative sexual potency, and prognosis. Whether and to what extent longer delays lead to poorer clinical consequences remain unknown. Under this background, we conducted this multicenter study in China to obtain statistical evidence about these issues for finally promoting patients to improve the ratio of early medical consultation.

## Methods

### Inclusion criteria

**Fig. 1:**
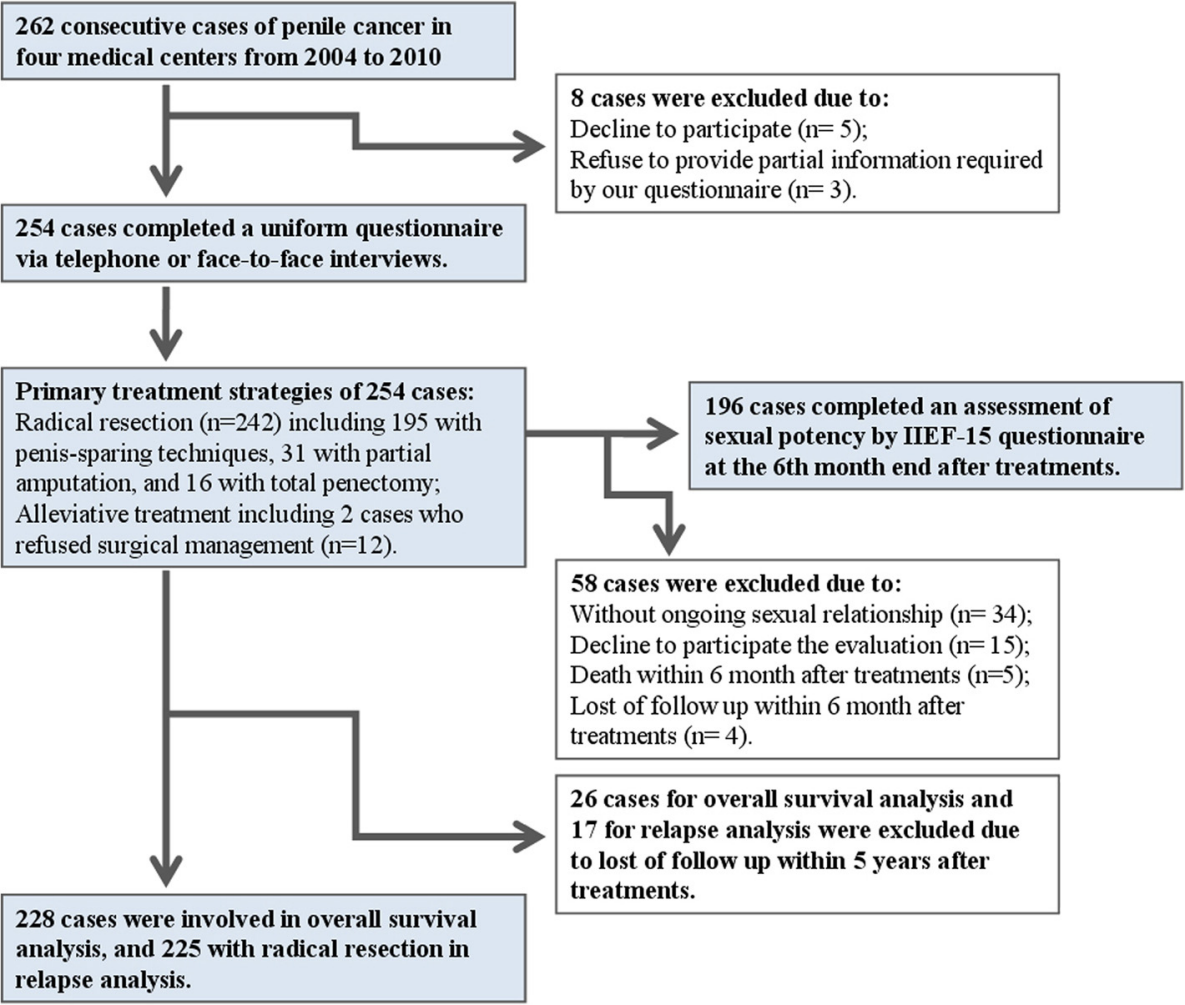
Flow diagram of patients involved in the study

From June 2004 to March 2010, 262 consecutive PC cases with various TNM stages received treatment at 4 medical centers. All patients were clearly diagnosed by histopathologic biopsy. Clinical stages were determined based on an overall consideration of physical examination in the inguinal area and a series of imaging examinations of the chest, abdomen, and pelvis. If necessary, a fine-needle aspiration biopsy of sentinel node under the guidance of ultrasound or an excisional biopsy of suspicious nodes was administered to confirm the stage [1, 7]. After a rigorous selection (Fig. 1), 254 cases (96.9 %) completed a uniform epidemiological questionnaire for investigating risk factors associated with patient’s delay; 196 (74.8 %) with ongoing sexual relationship accepted an assessment of sexual potency by the International Index of Erectile Function (IIEF)-15 questionnaire at the sixth-month end after treatments [8]; 228 (87.0 %) were involved in overall survival (OS) analysis and 225 (85.9 %) with radical resection in relapse analysis. Diverse reasons for patients excluded in each part of this study were shown in Fig. 1. The ethical committee of Nanjing Medical University approved the study in April 2004. Informed consent was obtained from all enrollees.

### Data collection

Data was collected via telephone or face-to-face interviews with the above-mentioned questionnaire. The questionnaire consisted of 85 closed-ended questions investigating 14 aspects of patients as follows: demographics, families, occupation, sexual activity, income, medical insurance, education, faith, lifestyle and habits, family history, health knowledge, initial symptom, relevant medical history, and treatment seeking. We defined patient’s delay as a time span from detecting initial symptom by patients themselves to first medical consultation [9]. Enrolled patients with a patient’s delay of >1 month were classified into delayed group, otherwise into undelayed group. Slight drinking was defined as drinking ≤400 ml/week (the integer of average) and heavy drinking was defined as >400 ml/week. Slight smoking was defined as smoking ≤15 cigarettes/day (the integer of average) and heavy smoking was defined as >15 cigarettes/day. Lesion characteristics, disease stage, and treatment strategies were extracted from medical records. To test the consistency of patients’ perception regarding symptoms and delay, answers were compared with data extracted from their medical records, and inconsistent data were further confirmed by querying patients again. Data for prognostic analyses was obtained via the combination of outpatient records, regular telephone follow-up, and patients reporting whenever necessary. Postoperative relapse was defined as any local, regional, or distant recurrence after radical resection, and new primary lesions were categorized into local recurrence.

### Statistical analysis

Descriptive statistics were used to describe demographics, patient’s delay, and reasons for delay. Univariate logistic regression analyses were used to assess crude associations between delay status (delayed or undelayed) and risk factors. Odds ratios (OR) with 95 % confidence intervals (CI) were calculated to evaluate risk effect of each variable. Variables with a *p* value <0.05 in univariate analyses were put into multivariate logistic regression models to identify those factors with the strongest independent effects. Chi-square test was used to investigate the impacts of diverse delay extents on clinical consequences.

## Results

### Patient’s delay and primary subjective reasons

**Fig. 2:**
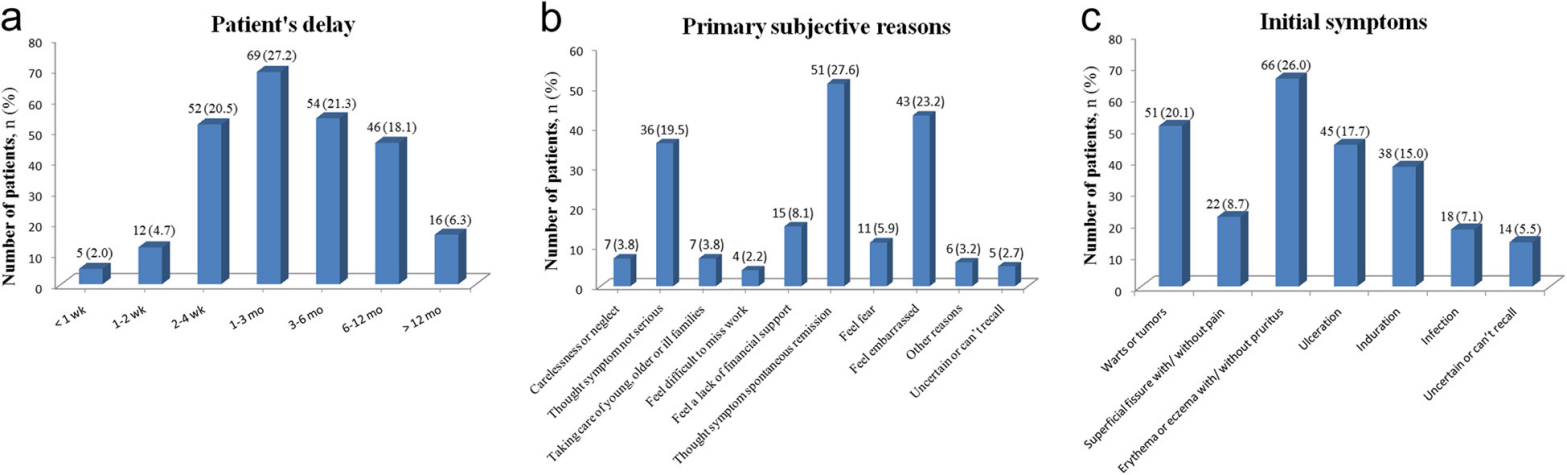
Distributions of patients’ numbers in time duration of patient’s delay (**a**), primary subjective reasons for delay (**b**), and initial symptoms (**c**)

In all of 254 enrollees, mean age at diagnosis was 57.3 years old (SD = 8.0) with the range from 38 to 78 years old. Mean delay for first medical consultation was 116 days (SD = 17.2), and the distribution of patients based on delayed time interval is shown in Fig. 2a. In total, 27.2 % of enrollees with a patient’s delay ≤4 weeks were considered undelayed while 72.8 % waited for >1 month before making first consultation, 45.7 % >3 months, and 24.4 % >6 months. In 185 delayed patients, 97.3 % of them gave a definite primary reason for their delay (Fig. 2b). Three most predominant reasons were the perceptions of “Thought symptom spontaneous remission” (27.6 %), “Feel embarrassed in describing the problem to medical practitioners” (23.2 %), and “Thought symptom not serious” (19.5 %).

### Initial symptoms

Total 240 of 254 enrollees (94.5 %) recalled their first symptoms associated with PC (Fig. 2c). Erythema or eczema with/without pruritus were the most common symptom (26.0 %) followed by warts or tumors (20.1 %), ulceration (17.7 %), induration (15.0 %), superficial fissure with/without pain (8.7 %), and infection (7.1 %).

### Risk factors associated with patient’s delay

Fifteen sociodemographic and 5 PC-related factors were investigated for the risk factors of patient’s delay by univariate analyses (Additional files 1 and 2: Tables S1 and S2). These unadjusted results showed that single, low frequency of sexual intercourse (0–1 time/month), poor education (≤ primary school), living in rural areas, never using Internet, heavy drinking alcohol, and without cancer family history were 7 sociodemographic risk factors significantly associated with delayed medical consultation (Additional file 1: Table S1). For analyzing the association between initial symptoms and patient’s delay, we set specific initial symptoms (warts or tumors) as a baseline to compare with other unspecific symptoms. Patients with initial symptoms such as erythema, eczema, or induration were found in a significant risk of delay. In addition, when setting patients without history of sexually transmitted infections (STIs) as a comparative baseline, an interesting result was found that the medical history of condyloma acuminatum displayed a significant protective effect for patient’s delay (Additional file 2: Table S2).

Table 1 exhibited adjusted results by a univariate model of 9 potential predictors identified by univariate analyses. Once balanced for other covariates, only single, living in rural areas, heavy drinking alcohol, aspecific initial symptoms such as erythema, eczema, or induration, and medical history of condyloma remained significant associations with patient’s delay (Table 1). Low frequency of sexual intercourse, poor education, never using Internet, and without cancer family history were proved not to be independent predictors.

**Table 1:** Multivariate logistic regression models of risk factors for delayed first medical consultation

Variables		Treatment seeking (%)		Multivariate analysis^a^	
		Undelayed (*n* = 69)	Delayed (*n* = 185)	OR (95 % CI)	*p* value
Marital status	Married	54 (78.3)	124 (67.0)	Reference	
	Cohabitation	4 (5.8)	6 (3.2)	0.439 (0.086–2.239)	0.322
	Single	11 (15.9)	55 (29.7)	2.372 (1.025–5.490)	0.044
Frequency of sexual intercourse/mo	≥5 times	15 (21.7)	26 (14.1)	Reference	
	2–4 times	43 (62.3)	104 (56.2)	1.102 (0.452–2.685)	0.831
	0–1 time	11 (15.9)	55 (29.7)	2.714 (0.918–8.022)	0.071
Education	≥ Graduate	13 (18.8)	21 (11.4)	Reference	
	Secondary school	37 (53.6)	84 (45.4)	1.238 (0.476–3.224)	0.662
	≤ Primary school	19 (27.5)	80 (43.2)	2.053 (0.741–5.687)	0.167
Place of residence	City or town	43 (62.3)	80 (43.2)	Reference	
	Rural area	26 (37.7)	105 (56.8)	2.524 (1.276–4.994)	0.008
Utilization of Internet	Almost everyday	19 (27.5)	38 (20.5)	Reference	
	Occasionally	38 (55.1)	81 (43.8)	0.553 (0.235–1.304)	0.176
	Never	12 (17.4)	66 (35.7)	1.866 (0.706–4.931)	0.208
Drinking alcohol habit, ml/week	Never or occasionally	19 (27.5)	32 (17.3)	Reference	
	Slight (≤400^a^)	43 (62.3)	119 (64.3)	1.380 (0.620–3.068)	0.430
	Heavy (>400)	7 (10.1)	34 (18.4)	3.574 (1.119–11.416)	0.032
Family history of cancer	Yes	21 (30.4)	34 (18.4)	Reference	
	None	48 (69.6)	151 (81.6)	1.999 (0.946–4.225)	0.070
Initial symptom	Warts or tumors	18 (26.1)	33 (17.8)	Reference	
	Superficial fissure with/without pain	7 (10.1)	15 (8.1)	1.223 (0.366–4.093)	0.744
	Erythema or eczema with/without pruritus	12 (17.4)	54 (29.2)	3.224 (1.165–8.925)	0.024
	Ulceration	15 (21.7)	30 (16.2)	0.840 (0.306–2.308)	0.736
	Induration	6 (8.7)	32 (17.3)	3.592 (1.068–12.084)	0.039
	Infection	8 (11.6)	10 (5.4)	0.682 (0.181–2.574)	0.573
	Uncertain or cannot recall	3 (4.3)	11 (5.9)	2.232 (0.481–10.356)	0.305
History of STIs	None or uncertain	34 (49.3)	116 (62.7)	Reference	
	Condyloma acuminatum	15 (21.7)	20 (10.8)	0.322 (0.128–0.812)	0.016
	Gonorrhea	7 (10.1)	24 (13.0)	1.218 (0.420–3.534)	0.716
	Syphilis	4 (5.8)	3 (1.6)	0.293 (0.048–1.809)	0.186
	Nongonococcal urethritis	6 (8.7)	16 (8.6)	0.872 (0.249–3.050)	0.830
	≥2 STIs	3 (4.3)	6 (3.2)	0.511 (0.091–2.873)	0.446

*STIs* sexually transmitted infections, *PC* penile carcinoma, *OR* odds ratios, *CI* confidence intervals

^a^Adjusted for all covariates in the table

### Negative consequences associated with patient’s delay

Impacts of diverse delay extents on lesion characteristics and primary treatment strategies were investigated based on 254 enrollees. When setting the undelayed group as a baseline, stratified analyses of delay extents exhibited that patients with delay of 3–6 months were in significant risks for bigger lesion size, more advanced stage of primary tumor, higher positive rate of regional lymph nodes, and lower penis-sparing rate under the premise of oncologic cure. Patients with delay >6 months were shown to be at higher risk as described above plus a significantly increased risk of metastasis. All *p* and OR values with 95 % CI were listed in Table 2.

**Table 2:** Impacts of diverse delayed extents on lesion characteristics and primary treatment strategies

Variables	Lesion size, cm (≤2 vs*.* >2^a^)		Primary tumor (Tis^b^/Ta/T1a vs. T1b–T4)		Lymph nodes (N0 vs*.* N1–N3)		Distant metastasis (M0 vs*.* M1)		Primary strategies (penis-sparing^c^ vs*.* others^d^)	
	OR (95 % CI)	*p* value	OR (95 % CI)	*p* value	OR (95 % CI)	*p* value	OR (95 % CI)	*p* value	OR (95 % CI)	*p* value
Undelayed (*n* = 69)	Reference		Reference		Reference		Reference		Reference	
Delay 1–3 months (*n* = 69)	1.432 (0.679–3.018)	0.342	1.502 (0.616–3.660)	0.369	1.920 (0.609–6.055)	0.259	1.015 (0.986–1.044)	1.000^e^	1.446 (0.543–3.850)	0.459
Delay 3–6 months (*n* = 54)	3.294 (1.533–7.077)	0.002	2.950 (1.227–7.092)	0.013	6.400 (2.192–18.689)	<0.001	1.059 (0.992–1.130)	0.082^e^	3.530 (1.376–8.891)	0.007
Delay >6 months (*n* = 62)	6.424 (2.993–13.787)	<0.001	3.726 (1.604–8.656)	0.002	7.549 (2.653–21.483)	<0.001	1.107 (1.021–1.201)	0.010^e^	4.497 (1.830–11.052)	0.001

*OR* odds ratios, *CI* confidence intervals, *Tis* carcinoma in situ

^a^The integer of average

^b^Penile carcinoma in situ includes erythroplasia and Bowen’s disease

^c^Penis-sparing includes penis-sparing surgery, micrographic surgery, and laser ablation or excision

^d^Others include partial amputation, total penectomy, and alleviative treatment

^e^Fisher exact test

Table 3 shows impacts of diverse delay extents on sexual potency at the sixth-month end after treatments. The IIEF-15 questionnaire disclosed that patients with delay of 3–6 months were in significant risks for inferior performance of all domains except sexual desire compared with undelayed patients. Patients with delay >6 months even displayed significantly higher risks than patients with delay of 3–6 months in all of 5 domains.

**Table 3:** Impacts of diverse delayed extents on the subjective evaluation of sexual potency at the 6th month end after treatments

IIEF-15 domains	Erectile function (19–30^a^ vs*.* 0–18)		Orgasmic function (7–10^a^ vs*.* 0–6)		Sexual desire (7–10^a^ vs*.* 0–6)		Intercourse satisfaction (10–15^a^ vs*.* 0–9)		Overall satisfaction (7–10^a^ vs*.* 0–6)	
	OR (95 % CI)	*p* value	OR (95 % CI)	*p* value	OR (95 % CI)	*p* value	OR (95 % CI)	*p* value	OR (95 % CI)	*p* value
Undelayed (*n* = 58)	Reference		Reference		Reference		Reference		Reference	
Delay 1–3 months (*n* = 60)	1.554 (0.650–3.712)	0.319	1.660 (0.755–3.650)	0.206	1.529 (0.508–4.608)	0.448	1.571 (0.702–3.519)	0.270	1.643 (0.708–3.812)	0.246
Delay 3–6 months (*n* = 37)	2.913 (1.151–7.371)	0.021	2.716 (1.135–6.498)	0.023	2.391 (0.756–7.565)	0.131	2.977 (1.233–7.190)	0.014	2.921 (1.177–7.250)	0.019
Delay >6 months (*n* = 41)	3.344 (1.358–8.231)	0.007	3.010 (1.288–7.034)	0.010	3.178 (1.067–9.466)	0.032	3.639 (1.541–8.594)	0.003	3.311 (1.368–8.009)	0.007

*IIEF* International Index of Erectile Function, *OR* odds ratios, *CI* confidence interval

^a^No or mild dysfunction for each domain of IIEF-15 [8]

In addition, we also analyzed the impacts of delay extents on OS and disease relapses at the second- and fifth-year ends after treatments. Only patients with delay >6 months were discovered in a significant risk for inferior survival at the second-year end compared with undelayed patients. Patient’s delay did not exhibit any significant impact on relapses after radical therapies and 5-year OS (Table 4).

**Table 4:** Impacts of diverse delayed extents on overall survival and disease relapses

Variables	Overall survival, year (≥2 vs*.* <2)		Overall survival, year (≥5 vs*.* <5)			Relapses within 2 years^a^ (none vs*.* relapses^b^)		Relapses within 5 years^a^ (none vs*.* relapses^b^)	
	OR (95 % CI)	*p* value	OR (95 % CI)	*p* value		OR (95 % CI)	*p* value	OR (95 % CI)	*p* value
Undelayed (*n* = 63)	Reference		Reference		Undelayed (*n* = 63)	Reference		Reference	
Delay 1–3 months (*n* = 64)	0.984 (0.134–7.209)	1.000^e^	1.309 (0.456–3.762)	0.616	Delay 1–3 months (*n* = 62)	0.803 (0.307–2.097)	0.653	0.870 (0.395–1.914)	0.728
Delay 3–6 months (*n* = 46)	2.905 (0.509–16.587)	0.238^e^	1.684 (0.564–5.034)	0.347	Delay 3–6 months (*n* = 46)	0.709 (0.242–2.081)	0.530	0.694 (0.286–1.689)	0.420
Delay >6 months (*n* = 55)	5.191 (1.053–25.598)	0.043^e^	2.476 (0.909–6.746)	0.070	Delay >6 months (*n* = 54)	0.945 (0.359–2.487)	0.909	0.714 (0.307–1.659)	0.433

*OR* odds ratios, *CI* confidence intervals

^a^After radical therapies

^b^Relapses includes local, regional, and distant recurrences, and new primary lesions are classified into local recurrence

^e^Fisher exact test

## Discussion

In the field of oncology, second best to prevention is early diagnosis and treatment. Patients treated at an early stage of malignant diseases commonly have better quality of life and longer survival [10, 11]. However, potential psychosocial factors and obscure initial symptoms can severely impede early clinical presentation of PC. After preliminary identification by univariate analyses, a multivariate model finally ascertained 4 risk factors (single, living in rural areas, heavy drinking alcohol, and aspecific initial symptoms) and 1 protective factor (medical history of condyloma) significantly associated with patient’s delay. Not surprisingly, the role of a partner was important in encouraging patients to timely seek treatment, which even could not be replaced by adult children. PC is a special disease that occurs in male private parts, so only their partners seem to be the optimal consulters before first medical consultation. Even facing medical practitioners, still 23.2 % of delayed patients in this study reported “Feel embarrassed” as the primary reason for their delay, which also partly explains why annual medical exam helps little to decrease the ratio of patient’s delay.

Another main reason causing the delay is aspecific initial symptoms of PC such as erythema, eczema, or induration, which easily mislead patients to think that their symptoms are not serious and will spontaneously resolve. Our investigation even discovered that good education, convenient Internet access, or warning from cancer family history contributed little for eliminating these cognitive barriers. Therefore, improving patient education on initial symptoms of PC is necessary in Chinese men of >40 years old, especially those living in rural area or with heavy drinking habit.

The most interesting and surprising finding in this study is that the medical history of condyloma was a significant protective factor for patient’s delay. However, it is well-known that a history of condyloma is associated with a 3–5-fold increased risk of PC [1, 12]. Three reasons may interpret this unexpected phenomenon: (1) Patients with a history of condyloma have more opportunities to obtain information about PC; (2) mistakenly regarding initial lesions of PC as the recurrence of previous condyloma instead of other inessential benign diseases contributes to timely treatment seeking; (3) the previous experience of curing condyloma helps patients overcome the influence of adverse psychological factors such as “Feel embarrassed or fear”. Moreover, phimosis and smoking are also risk factors of PC [1, 13], but none of them were found significantly associated with patient’s delay.

In addition, 24.4 % of enrollees in this cohort delayed treatment seeking for >6 months, which was much lower than that in two Swedish studies [3, 4]. We supposed that it should be attributed to the extremely low expenses for outpatient consultation (usually <2 dollars/visit) in most areas of China based on the following two facts. One was confirmed by univariate analyses that low family income and lack of medical insurance were not the significant risk factors for delay; the other was that only 8.1 % of delayed patients reported “Feel a lack of financial support” as a primary reason. Becker found that financial concerns stopped uninsured people from seeking care unless they had severe pain or believed that they would die [14]. Therefore, reducing the charge for first medical consultation might contribute to improving the ratio of early clinical presentation of PC, which even can be extrapolated to the whole field of oncology.

Another main finding of this study is that delay >3 months will lead to significantly higher risks for large lesion size and advanced TNM classification. These adverse clinical characteristics significantly decrease the rate of organ sparing under the premise of oncologic cure. However, competent performance for sexual intercourse is extremely important for males [15]. Partial or total amputation inevitably leads to a devastating effect on penile appearance and function, followed by strong impacts on patient’s psychosexual and psychosocial well-being [16, 17]. Penis-sparing techniques obviously allow a better quality of life than amputation but only applicable for superficial PC [1]. Therefore, to minimize patient’s delay is the key of resuming superior sexual function.

Somewhat surprising is that patient’s delay did not impact on postoperative relapses and 5-year OS, and only patients with delay >6 months showed significantly inferior 2-year OS. It can be interpreted by the following three facts: (1) Amputation surgeries offer excellent oncologic control for patients with stage T1b–T3 [16, 18]; (2) nearly 100 % of distant recurrences, which can cause death, occur during the first 2 years of follow-up [19]; (3) patients with delay >6 months have a significant risk of metastasis in our cohort.

This current study has some limitations. First, the time span of delay is impossible to be exactly self-reported by patients, and the investigation was inevitably hampered by recall bias as well as patient’s perception. However, we believe that the bias does not likely play a significant role because the consistency of data between patients reporting and their medical records has been checked carefully. Second, patient’s delay is not the unique delay affecting patient’s prognosis and functional restoration. Doctor’s delay, defined as time span from first medical consultation to the beginning of correct treatment based on accurate diagnosis, also strongly impacts on the clinical consequences [20]. Therefore, a further study focusing on delay after first medical presentation is warranted.

## Conclusions

Single, living in rural areas, heavy drinking alcohol, and aspecific initial symptoms are significant risk factors of PC associated with patient’s delay. Delay >3 months may lead to significant risks for adverse clinical characteristics, low penis-sparing rate, and poor sexual function restoration. Although patient’s delay did not impact on postoperative relapses, delay >6 months may have significantly inferior 2-year OS. Minimizing patient’s delay is the key to avoid amputation and resume superior sexual potency; thus, improving patient education on initial symptoms of PC is necessary in men of >40 years old.

## Additional files

Additional file 1: Table S1. Comparisons of sociodemographic factors between delayed and undelayed patients. (DOC 106 kb) Additional file 2: Table S2. Comparisons of penile cancer-related factors between delayed and undelayed patients. (DOC 72 kb)
