# Supplementary material for: Risk factors and negative consequences of patient’s delay for penile carcinoma
Source: World J Surg Oncol. 2016 Apr 27;14:124. doi: 10.1186/s12957-016-0863-z (PMC4848776; doi:10.1186/s12957-016-0863-z)
Supplement: Additional file 1: Table S1. — Comparisons of sociodemographic factors between delayed and undelayed patients. (DOC 106 kb) [file 12957_2016_863_MOESM1_ESM.doc]

**Supplemental Table 1. Comparisons of** **sociodemographic** **factors between** **delayed and undelayed patients**

| Variables |  | Treatment seeking (%) | |  | Univariate analysis | |
| --- | --- | --- | --- | --- | --- | --- |
|  |  | Undelayed (n =69) | Delayed (n=185) |  | OR (95% CI) | *p* value |
| Age at diagnosis, yr | ≤ 57^a^ | 33 (47.8) | 101 (54.6) |  | Reference |  |
|  | > 57 | 36 (52.2) | 84 (45.4) |  | 0.762 (0.438-1.327) | 0.336 |
| Marital status | Married | 54 (78.3) | 124 (67.0) |  | Reference |  |
|  | Cohabitation | 4 (5.8) | 6 (3.2) |  | 0.653 (0.177-2.409) | 0.501^e^ |
|  | Single | 11 (15.9) | 55 (29.7) |  | 2.177 (1.058-4.482) | 0.032 |
| Living with children of > 18 yr old | Yes | 31 (44.9) | 77 (41.6) |  | Reference |  |
|  | No | 38 (55.1) | 108 (58.4) |  | 1.144 (0.655-1.998) | 0.635 |
| Frequency of sexual intercourse, /mo | ≥ 5 times | 15 (21.7) | 26 (14.1) |  | Reference |  |
|  | 2-4 times | 43 (62.3) | 104 (56.2) |  | 1.395 (0.647-2.890) | 0.369 |
|  | 0-1 time | 11 (15.9) | 55 (29.7) |  | 2.885 (1.164-7.146) | 0.020 |
| Occupation | Retirement | 23 (33.3) | 54 (29.2) |  | Reference |  |
|  | Informal employment | 25 (36.2) | 60 (32.4) |  | 1.022 (0.520-2.008) | 0.949 |
|  | Formal employment | 21 (30.4) | 71 (38.4) |  | 1.440 (0.723-2.869) | 0.299 |
| Monthly family income, dollar | >1000 | 16 (23.2) | 45 (24.3) |  | Reference |  |
|  | 500-1000 | 35 (50.7) | 88 (47.6) |  | 0.894 (0.447-1.786) | 0.751 |
|  | < 500 | 18 (26.1) | 52 (28.1) |  | 1.027 (0.470-2.247) | 0.947 |
| Medical insurance | Insured | 28 (40.6) | 68 (36.8) |  | Reference |  |
|  | Uninsured | 41 (59.4) | 117 (63.2) |  | 1.175 (0.667-2.069) | 0.576 |
| Annual medical exam | Yes | 37 (53.6) | 89 (48.1) |  | Reference |  |
|  | No | 32 (46.4) | 96 (51.9) |  | 1.247 (0.717-2.171) | 0.434 |
| Education | ≥ Graduate | 13 (18.8) | 21 (11.4) |  | Reference |  |
|  | Secondary school | 37 (53.6) | 84 (45.4) |  | 1.405 (0.636-3.104) | 0.399 |
|  | ≤ Primary school | 19 (27.5) | 80 (43.2) |  | 2.607 (1.110-6.120) | 0.025 |
| Faith | No | 62 (89.9) | 161 (87.0) |  | Reference |  |
|  | Buddhism or others | 4 (5.8) | 14 (7.6) |  | 1.348 (0.427-4.253) | 0.786 ^e^ |
|  | Christianity | 3 (4.3) | 10 (5.4) |  | 1.284 (0.342-4.820) | 1.000 ^e^ |
| Place of residence | City or town | 43 (62.3) | 80 (43.2) |  | Reference |  |
|  | Rural area | 26 (37.7) | 105 (56.8) |  | 2.171 (1.231-3.828) | 0.007 |
| Utilization of internet | Almost every day | 19 (27.5) | 38 (20.5) |  | Reference |  |
|  | Occasionally | 38 (55.1) | 81 (43.8) |  | 1.066 (0.544-2.087) | 0.853 |
|  | Never | 12 (17.4) | 66 (35.7) |  | 2.750 (1.204-6.279) | 0.014 |
| Drinking alcohol habit, ml/ wk | Never or occasionally | 19 (27.5) | 32 (17.3) |  | Reference |  |
|  | Slight (≤ 400^a^) | 43 (62.3) | 119 (64.3) |  | 1.643 (0.844-3.199) | 0.142 |
|  | Heavy (> 400) | 7 (10.1) | 34 (18.4) |  | 2.884 (1.069-7.777) | 0.033 |
| Smoking habit, cigarettes/ d | Never or occasionally | 24 (34.8) | 53 (28.6) |  | Reference |  |
|  | Slight (≤ 15^a^) | 25 (36.2) | 70 (37.8) |  | 1.268 (0.653-2.463) | 0.483 |
|  | Heavy (> 15) | 20 (29.0) | 62 (33.5) |  | 1.404 (0.699-2.820) | 0.340 |
| Family history of cancer | Yes | 21 (30.4) | 34 (18.4) |  | Reference |  |
|  | None | 48 (69.6) | 151 (81.6) |  | 1.943 (1.031-3.661) | 0.038 |

OR = Odds ratios; CI = confidence intervals.

^a^ the integer of average.

^e^ Fisher exact test.
