# Supplementary material for: Risk factors and negative consequences of patient’s delay for penile carcinoma
Source: World J Surg Oncol. 2016 Apr 27;14:124. doi: 10.1186/s12957-016-0863-z (PMC4848776; doi:10.1186/s12957-016-0863-z)
Supplement: Additional file 2: Table S2. — Comparisons of penile cancer-related factors between delayed and undelayed patients. (DOC 72 kb) [file 12957_2016_863_MOESM2_ESM.doc]

**Supplemental Table 2****. Comparisons of** **penile cancer-related factors between delayed and undelayed patients**

| Variables |  | Treatment seeking (%) | |  | Univariate analysis | |
| --- | --- | --- | --- | --- | --- | --- |
|  |  | Undelayed (n=69) | Delayed (n=185) |  | OR (95% CI) | *p* value |
| Initial symptom | Warts or tumors | 18 (26.1) | 33 (17.8) |  | Reference |  |
|  | Superficial fissure with/ without pain | 7 (10.1) | 15 (8.1) |  | 1.169 (0.403-3.391) | 0.774 |
|  | Erythema or eczema with/ without pruritus | 12 (17.4) | 54 (29.2) |  | 2.455 (1.050-5.738) | 0.036 |
|  | Ulceration | 15 (21.7) | 30 (16.2) |  | 1.091 (0.469-2.540) | 0.840 |
|  | Induration | 6 (8.7) | 32 (17.3) |  | 2.909 (1.024-8.264) | 0.040 |
|  | Infection | 8 (11.6) | 10 (5.4) |  | 0.682 (0.229-2.034) | 0.491 |
|  | Uncertain or can’t recall | 3 (4.3) | 11 (5.9) |  | 2.000 (0.493-8.109) | 0.520^e^ |
| Lesion location | Glans or coronary sulcus | 34 (49.3) | 83 (44.9) |  | Reference |  |
|  | Scapus penis or foreskin | 19 (27.5) | 54 (29.2) |  | 1.164 (0.603-2.247) | 0.650 |
|  | Both | 16 (23.2) | 48 (25.9) |  | 1.229 (0.615-2.456) | 0.559 |
| History of circumcision | Circumcised | 7 (10.1) | 16 (8.6) |  | Reference |  |
|  | Uncircumcised | 62 (89.9) | 169 (91.4) |  | 1.193 (0.468-3.037) | 0.712 |
| History of STIs | None or uncertain | 34 (49.3) | 116(62.7) |  | Reference |  |
|  | Condyloma acuminatum | 15 (21.7) | 20 (10.8) |  | 0.414 (0.192-0.891) | 0.022 |
|  | Gonorrhoea | 7 (10.1) | 24 (13.0) |  | 1.064 (0.424-2.673) | 0.895 |
|  | Syphilis | 4 (5.8) | 3 (1.6) |  | 0.233 (0.050-1.089) | 0.068^e^ |
|  | Nongonococcal urethritis | 6 (8.7) | 16 (8.6) |  | 0.828 (0.301-2.272) | 0.713 |
|  | ≥ 2 STIs | 3 (4.3) | 6 (3.2) |  | 0.621 (0.148-2.608) | 0.512^e^ |
| Previously heard of PC | Never | 29 (42.0) | 97 (52.4) |  | Reference |  |
|  | Yes | 40 (58.0) | 88 (47.6) |  | 0.658 (0.376-1.150) | 0.140 |

STIs = sexually transmitted infections; PC = penile squamous cell carcinoma; OR = Odds ratios; CI = confidence intervals.

^e^ Fisher exact test.
